# HEIH Promotes Malignant Progression of Gastric Cancer by Regulating STAT3-Mediated Autophagy and Glycolysis

**DOI:** 10.1155/2022/2634526

**Published:** 2022-07-27

**Authors:** Huiqing Zhang, Xiaohua Shen, Shuping Xiong, Lixiang Peng, Wenli Mai, Longxiang Xin

**Affiliations:** ^1^The Medical College of Nanchang University, Nanchang, Jiangxi 330006, China; ^2^Department of Gastrointestinal Medical Oncology, Jiangxi Cancer Hospital, Nanchang, Jiangxi 330029, China

## Abstract

To study the clinical value of HEIH hyperexpression in gastric cancer and the molecular mechanism of promoting malignant proliferation of gastric cancer cells, qRT-PCR was used to detect the expression of HEIH in gastric cancer and nontumor gastric tissues. HEIH interference sequence was constructed to downregulate HEIH expression in MGC-803 and BGC-823 cell lines. CCK8, clonogenesis, and Transwell assay were used to detect the effects of HEIH on proliferation and invasion of tumor cells. The protein levels of STAT3, p-STAT3, P62, and LC3 were detected by Western blotting. The results showed that HEIH was highly expressed in gastric cancer (*P* < 0.01). Interference of HEIH expression in MGC-803 and BGC-823 cells reduced the proliferation and invasion of gastric cancer cells, and the results were statistically significant (*P* < 0.05). HEIH acts as a miRNA sponge for miR-4500. HEIH promotes gastric cancer development by inhibiting miR-4500. STAT3 is a downstream target of miR-4500. HEIH inhibits autophagy and promotes glycolysis. In conclusion, HEIH is highly expressed in gastric cancers. HEIH promotes malignant proliferation and development of gastric cancer cells. HEIH may be a new candidate site for pathological diagnosis and molecular drug therapy for future clinical treatment of gastric cancer.

## 1. Introduction

Gastric cancer is one of the most common malignancies in the world and is the third leading cause of cancer-related deaths, with more than 1 million new cases per year. The 5-year overall survival rate of patients with localized, early gastric cancer is greater than 60%, and the 5-year overall survival rate of patients with gastric cancer with local and distant metastasis has substantially decreased to 30% and 5%, respectively [1–3]. The early clinical symptoms of gastric cancer are concealed and atypical, so more than 60% of patients are diagnosed with local or distant metastasis. Surgical resection is the best treatment option for patients with early gastric cancer; chemotherapy is the most important treatment for patients who cannot undergo surgical resection or have advanced metastasis [4]. However, patients with gastric cancer respond very poorly to chemotherapy due to intrinsic or acquired resistance, which becomes the most common cause of treatment failure. Low early diagnosis rate and chemoresistance are the main reasons for poor prognosis of gastric cancer [5].

Long noncoding RNA (lncRNA) is a class of noncoding RNA (ncRNA) with a length of more than 200 nucleotides that was discovered in recent years in the study of human genome [1]. lncRNAs do not have the ability to encode proteins but regulate gene expression through various pathways in the form of RNA, including tumor genes [2]. Relevant lncRNAs have been found to play a key regulatory role in the occurrence and development of malignant gastric cancer [4]. It has been reported that HEIH enhances paclitaxel tolerance in endometrial cancer cells by activating the MAPK signaling pathway [5]. In addition, lncRNA-HEIH in serum and exosomes may serve as a potential biomarker for HCV-associated hepatocellular carcinoma [6]. In colorectal cancer, HEIH promotes the development of colorectal cancer by regulating the miR-939/Bcl-XL axis [7]. However, the relationship between HEIH and EMT has not been reported.

Studies have confirmed that miR-4500 plays a similar role as “tumor suppressor gene” in the progression of thyroid cancer, non-small-cell lung cancer, colorectal cancer, and other tumors [8–10]. However, the expression pattern of miR-4500 in gastric cancer and whether it plays the function of “tumor suppressor gene” in gastric cancer remain unclear.

Signal transduction and transcription activation factor 3 (STAT3) is an important intracellular signal transduction factor, which is involved in various physiological and pathological processes of cells [11]. Autophagy is a mode of cell self-degradation, which can provide energy for cell metabolism and maintain the homeostasis of the intracellular environment [12, 13]. STAT3 and autophagy play important roles in tumor genesis and development [14]. Previous studies have shown that STAT3 and autophagy levels are altered and abnormal in a variety of tumors, and STAT3 and autophagy may play a synergistic or antagonistic role in different stages of tumor pathological changes, thus promoting or inhibiting the occurrence and development of tumors [15, 16].

The objective of this study was to determine the abnormal expression of HEIH in human gastric cancer and to investigate the relationship between HEIH differential expression and malignant degree of gastric cancer. To further study the function of malignant proliferation of HEIH tumor cells, we hope to provide a new theoretical basis for exploring the molecular mechanism of malignant proliferation of brain gastric cancer. At the same time, provide new research evidence for molecular therapy of clinical gastric cancer.

## 2. Methods

### 2.1. Gastric Cancer Tissue Collection

Thirty clinical gastric cancer samples and paracancer control samples were collected between September 2019 and December 2020. The specimens were obtained from patients who had been diagnosed with gastric cancer and underwent surgery. No local or systemic therapy was performed before surgery. All patients signed informed consent. Specimens were assessed by the pathology department. All specimens were frozen in liquid nitrogen immediately after excision. Afterward, they were stored in an -80°C refrigerator for later use. This project is approved by the Ethics Research Committee of Cancer Hospital of Nanchang University.

### 2.2. Cell Culture

MGC-803 is a human gastric cancer cell. The morphological characteristics are epithelial-like and adherent growth. MGC-803 was established in a 53-year-old man with primary poorly differentiated myxoid adenocarcinoma of the stomach. BGC-823 is a human gastric adenocarcinoma cell (poorly differentiated). BGC-823 cells were derived from a 62-year-old patient with gastric cancer (undifferentiated adenocarcinoma). BGC-823 cells could express CEA. In this study, MGC-803 and BGC-823 cells were cultured in RPMI medium containing 10%FBS. Double antibody (100 U/mL penicillin and 100 mg/mL streptomycin) was added to the medium. Culture conditions: constant temperature incubator, 37°C, 5% CO_2_. According to the cell growth condition, the medium was changed from 1 to 3 days, and the culture was carried out when the culture dish was about 80% to 90% full of cell melt growth.

### 2.3. Cell Transfection

The interference sequences of HEIH (si-HEIH 1#, si-HEIH 2#) and out-of-order control (si-NC) were purchased from GenePharma. About 2 × 10^5^ cells/wells of MGC-803 and BGC-823 cells were inoculated into 6-well culture plates. 100 pmol siRNA and si-NC or 4 *μ*g pcDNA and pcDNA-STAT3 were diluted with 200 *μ*L Opti-MEM and incubated at room temperature for 5 min. The incubated liposomes were mixed with plasmids or siRNA diluents, gently blown and mixed, and incubated at room temperature for 20 min. Then, drop evenly into the 6-well culture plate with 1.6 Opti-MEM in advance. Culture at 37°C for 6 h in the incubator with 5% CO_2_. Change the medium completely. Continue to cultivate. After transfection 48 h, cells were collected and total RNA was extracted for qRT-PCR analysis and other subsequent experiments.

### 2.4. Subcellular Localization of HEIH Was Verified by RNA Cytoplasmic Nucleus Isolation

Cells were collected and total cell RNA, cytoplasmic RNA, and nuclear RNA were extracted in strict accordance with the operation instructions of the kit. The expression of HEIH was detected by Takara reverse transcription reagent and qRT-PCR. *β*-Actin was used as cytoplasmic reference and U6 as nuclear reference.

### 2.5. Cell Proliferation Activity Was Detected by CCK8

The cells were cultured in 6-well plates, and their density was about 60%. The cells were collected 24 h after transfection, and the artificial cell count was 1000 cells/well, and the cells were inoculated into 96-well culture plates. Each sample is set with 6 multiple holes. After about 80% of the inoculated cells adhered to the wall, the cells continued to be cultured for 12 h. Add 20 *μ*L CCK8 reaction solution directly. It was incubated at 37°C for 2 h away from light. The absorbance at 490 nm was measured by a microplate reader. The results were repeated three times.

### 2.6. Clone Formation Experiment

The cells were inoculated on 6-well plates and cultured to an appropriate density of 60% before transfection. Cell counts were collected 24 h after transfection. The cells were seeded into 6-well plates at an appropriate density (about 500) and placed in a 5% CO_2_ incubator at 37°C for further culture for 14 days. Add medium 1 time if necessary. Culture was terminated when clones were visible in the petri dish. PBS was gently cleaned, and 1 mL methanol was added for 60 min. 1 mL 0.1% crystal violet was added and dyed for 60 min. No enzyme washing to remove residual dyeing solution, air drying. The number of clones with more than 10 cells was counted under a microscope (low magnification). Clone formation rate was calculated.

### 2.7. Transwell Experiment

The density of transfected cells was adjusted to 3 × 10^6^ cells/mL in serum-free medium, and 2 × 10^4^ cells were injected into the upper compartment. 500 *μ*L RPMI 1640 medium containing 10% FBS was injected into the lower chamber. It was placed in an incubator and cultured for 12 h. A cotton swab gently wipes away the superior ventricle cells that have not penetrated the submembrane, methanol fixation for 5 min, 0.1% crystal violet staining for 5 min, water cleaning. Photographs were taken under an inverted microscope. The experiment was repeated three times.

### 2.8. qRT-PCR

Collect each group of cells, and add TRIzol reagent to extract total RNA from cells. According to the reverse transcription kit, reverse transcribe to synthesize cDNA. The primers of the target gene and the internal reference *β*-actin gene were designed and synthesized by BGI. miR-4500 upstream primer: 5′-GGGGTGAGGTAGTAG-3′, downstream primer: 5′-CAGTGCGTGTCGTGGAGT-3′. U6 upstream primer: 5′-CTCGCTTCGGCAGCACA-3′, downstream primer: 5′-AACGCTTCACGAATTTGCGT-3′. STAT3 upstream primer: 5′-GGACATCAGCGGTAAGACCC-3′, downstream primer: 5′-CCTGGGTCAGCTTCAGGATG-3′. Reaction conditions: 95°C predenaturation for 30 s; 95°C denaturation for 5 s, 60°C annealing for 30 s, 72°C extension for 30 s, 40 cycles; 72°C final extension for 7 minutes. The 2^-*ΔΔ*Ct^ method was used to calculate the mRNA expression level of each gene.

### 2.9. Bioinformatics Analysis

Bioinformatics software TargetScan predicted that the target gene of miR-4500 might be STAT3. Bioinformatics software Starbase predicted that the target gene of HEIH might be miR-4500.

### 2.10. Dual Luciferase Reporter Gene

The transfected cells were randomly divided into 4 groups: wild-type+miR-4500 group (transformed into wild-type plasmid+miR-4500), wild-type+miR-NC group (transformed into wild-type plasmid+miR-NC), mutant+miR-4500 group (transformed into mutant plasmid+miR-4500), and mutant+miR-NC group (transformed into mutant plasmid+miR-NC). Use dual luciferase reporter test kit for detection. The firefly luciferase activity and Renilla luciferase activity of the 4 groups of cells were analyzed. The experiment was repeated three times.

### 2.11. FISH

Cells in good growth state and in logarithmic growth phase were taken and placed into a 10 mm × 10 mm glass slide in a 24-well culture plate. The cells were inoculated into 24 empty plates at a density of 5 × 10^3^ cells per well. After 24 h, the supernatant was removed and 4% paraformaldehyde was fixed. 0.1% Triton X-100 permeable, prehybridization solution prehybridized at 37°C. The probes were hybridized at 42°C for 16-20 h. Then, rinse with 2× SSC and drop DAPI into the section hybridization area for 10 min. PBS was cleaned and observed under fluorescence microscope.

### 2.12. Western Blot

Cells were taken from each group, and total protein was extracted by adding protein lysate. Protein concentration was quantified by BCA method. 20 *μ*g protein was extracted and subjected to 8% SDS-PAGE. The isolated protein gel was transferred to the NC membrane. 5% skim milk powder was sealed for 120 min, then primary antibody (diluted at 1 : 1000) was added at 4°C to seal overnight, and TBST was washed for 5 min × 3 times. Diluted secondary antibody was added and incubated at room temperature for 1 h and then washed with TBST, exposure, development, photography. The gray value of strip was determined by ImageJ image analysis software. The ratio of gray value of each target protein to GAPDH gray value was used as the relative expression of target protein.

### 2.13. Statistical Analysis

SPSS 20.0 was used for statistical analysis. Results were shown by mean ± standard deviation of 3 experiments. The difference between the two groups was tested by two-tailed Student's ST. One-way ANOVA was used for comparison between multiple groups. The statistical results were represented by *P* value, ^∗^*P* < 0.05, which was statistically significant, and ^∗∗^*P* < 0.01, which was statistically significant.

## 3. Results

### 3.1. HEIH Is Highly Expressed in Gastric Cancer Tissues

**Figure 1:**
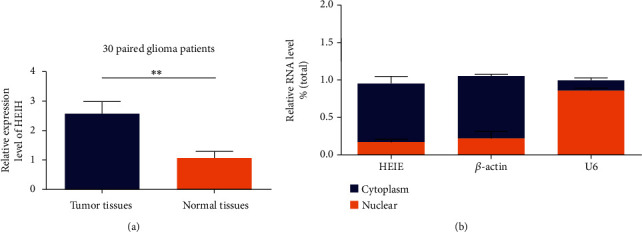
Identification of HEIH in gastric cancer. (a) Detection of HEIH expression in gastric cancer tissues relative to matched normal tissues (*n* = 30). (b) Analyze the expression of HEIH in the cytoplasm and nucleus, respectively. ^∗∗^*P* < 0.01.

qRT-PCR was used to analyze the expression of HEIH in 30 cases of gastric cancer and nontumor brain tissues. The results showed that HEIH expression was upregulated in gastric cancer tissues compared with adjacent tissues (Figure 1(a)), and the difference was statistically significant. The expression of HEIH in the cytoplasm and nucleus was analyzed. The results showed that HEIH was mainly distributed in the cytoplasm (Figure 1(b)).

### 3.2. Downregulation of HEIH Inhibits Malignant Proliferation, Migration, and Invasion of Gastric Cancer Cell Lines

**Figure 2:**
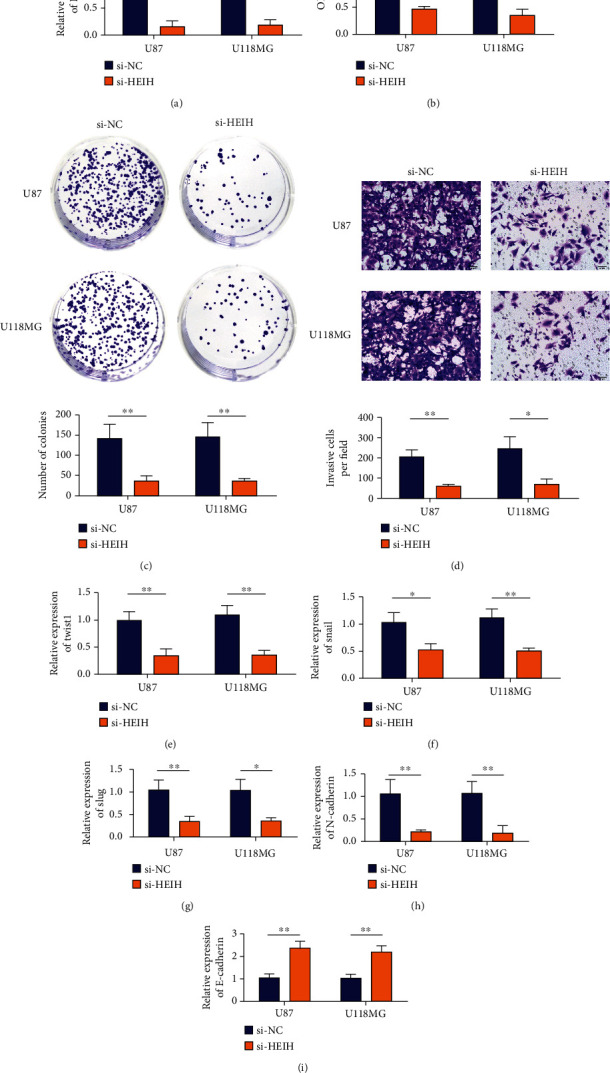
In vitro, HEIH promotes the proliferation, migration, and invasion of gastric cancer cells. (a) Transfection efficiency test results. (b) CCK8 method to detect the proliferation of MGC-803 and BGC-823 cell lines transfected with HEIH siRNA. (c) Assess cell proliferation through colony formation experiments. (d) Assess aggressiveness through Transwell analysis. (e) qRT-PCR detects the expression level of Twist1 in MGC-803 and BGC-823 cell lines after transfection. (f) qRT-PCR detects the expression level of Snail in MGC-803 and BGC-823 cell lines after transfection. (g) qRT-PCR detects the expression level of Slug in MGC-803 and BGC-823 cell lines after transfection. (h) qRT-PCR detects the expression level of N-cadherin in MGC-803 and BGC-823 cell lines after transfection. (i) qRT-PCR detects the expression level of E-cadherin in MGC-803 and BGC-823 cell lines after transfection. ^∗^*P* < 0.05 and ^∗∗^*P* < 0.01.

siRNA HEIH transfection in MGC-803 and BGC-823 cells was detected by qRT-PCR. The results showed that HEIH expression was decreased in siRNA transfected cells compared with the control group (Figure 2(a)). In MGC-803 and BGC-823 cell lines, the absorbance value of CCK8 at 490 nm was detected after transfection with si-HEIH. Compared with the control group (si-NC), the proliferation activity of MGC-803 and BGC-823 cells was significantly decreased after transfection with si-HEIH (Figure 2(b)). Cloning experiments suggested that knockdown HEIH significantly inhibited the clonogenesis of MGC-803 and BGC-823 cells (Figure 2(c)). Transwell experiment results showed that the number of invasive cells in the interference group was significantly reduced (Figure 2(d)), and the difference between si-HEIH and si-NC was statistically significant. The results of EMT-related markers showed that the expression of Twist1, Snail, Slug, and N-cadherin mRNAs in MGC-803 and BGC-823 cells was decreased by HEIH lowering (Figures 2(e)–2(h)), while the expression of E-cadherin mRNA was upregulated (Figure 2(i)).

### 3.3. Matching Sequence of HEIH and miR-4500

**Figure 3:**
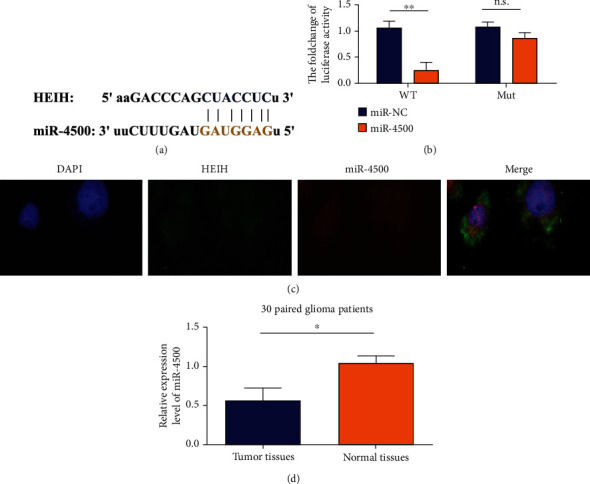
HEIH as a miRNA sponge for miR-4500. (a) miR-4500 is a potential target of HEIH predicted by the Starbase website. (b) The dual luciferase reporter gene method is used to detect the relative activity of luciferase in BGC-823 cells cotransfected with HEIH-WT/MUT and miR-4500 mimics. (c) FISH detects HEIH and miR-4500 in BGC-823 cell line. (d) Detect the expression level of miR-4500 in gastric cancer tissues relative to matching normal tissues by qRT-PCR (*n* = 30). ^∗^*P* < 0.05 and ^∗∗^*P* < 0.01.

Starbase, a bioinformatics software, predicted that miR-4500 might be the target gene of HEIH (Figure 3(a)). As can be seen from Figure 3(b), the relative luciferase activity of the wild-type+miR-4500 group was significantly lower than that of the wild-type+miR-NC group. These results indicate that miR-4500 can effectively inhibit the activity of wild-type plasmid luciferase. The relative luciferase activity of the mutant+miR-4500 group was significantly higher than that of the wild-type+miR-4500 group, indicating that miR-4500 could not inhibit the mutant plasmid luciferase activity. Fluorescence in situ hybridization was used to detect the colocalization of HEIH and miR-4500 in BGC-823 cell line. The results showed that HEIH and miR-4500 were colocated in the cytoplasm (Figure 3(c)). qRT-PCR was used to analyze the expression of miR-4500 in 30 gastric cancer tissues and nontumor adjacent tissues. The results showed that compared with the adjacent tissues, the expression of miR-4500 in gastric cancer tissues was decreased (Figure 3(d)), with statistically significant difference.

### 3.4. HEIH Promotes Gastric Cancer Development by Inhibiting miR-4500

**Figure 4:**
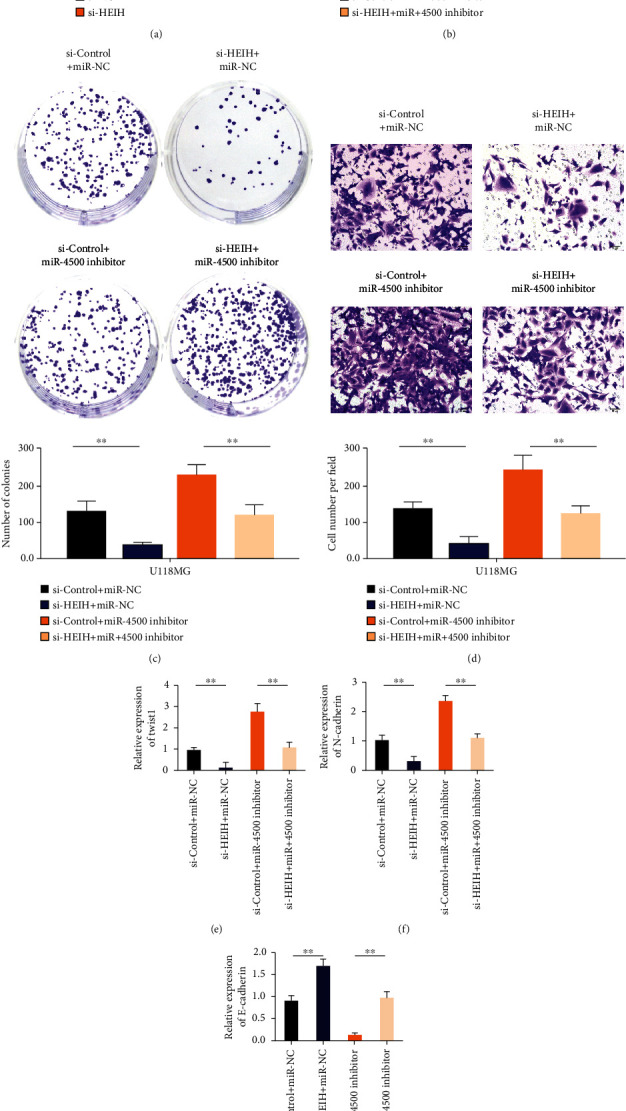
HEIH promotes the development of gastric cancer by inhibiting miR-4500. (a) Knockdown of HEIH can upregulate the expression of miR-4500. (b) Analysis of cell proliferation ability after cotransfection with HEIH-specific siRNA or miR-4500 inhibitor. (c) After cotransfection with HEIH-specific siRNA or miR-4500 inhibitor, cell colony formation experiment. (d) Transwell cell invasion test after cotransfection with HEIH-specific siRNA or miR-4500 inhibitor. (e) qRT-PCR detects the expression level of Twist1 in the cell line after cotransfection. (f) qRT-PCR detects the expression level of N-cadherin in the cell line after cotransfection. (g) qRT-PCR detects the expression level of E-cadherin in the cell line after cotransfection. ^∗^*P* < 0.05 and ^∗∗^*P* < 0.01.

Real-time PCR results showed that the expression level of miR-4500 in the si-HEIH group was significantly higher than that in the si-NC group, and the difference between the two groups was statistically significant (Figure 4(a)). CCK8 results showed that compared with the si-HEIH+miR-NC group, the cell activity of the si-HEIH+miR-4500 inhibitor cotransfection group was enhanced (Figure 4(b)). Compared with the si-HEIH+miR-NC group, cell clonal formation and invasion ability were enhanced in the si-HEIH+miR-4500 inhibitor cotransfection group (Figures 4(c) and 4(d)). After HEIH silencing, the expression of Twist1 and N-cadherin decreased. After transfection with si-HEIH+miR-4500 inhibitor, the expression levels of Twist1 and N-cadherin were upregulated (Figures 4(e) and 4(f)). The expression of E-cadherin increased after HEIH silencing. After transfection with si-HEIH+miR-4500 inhibitor, the expression of E-cadherin was decreased (Figure 4(g)).

### 3.5. Pairing Sequence of the 3′ Untranslated Region of STAT3 Gene with miR-4500

**Figure 5:**
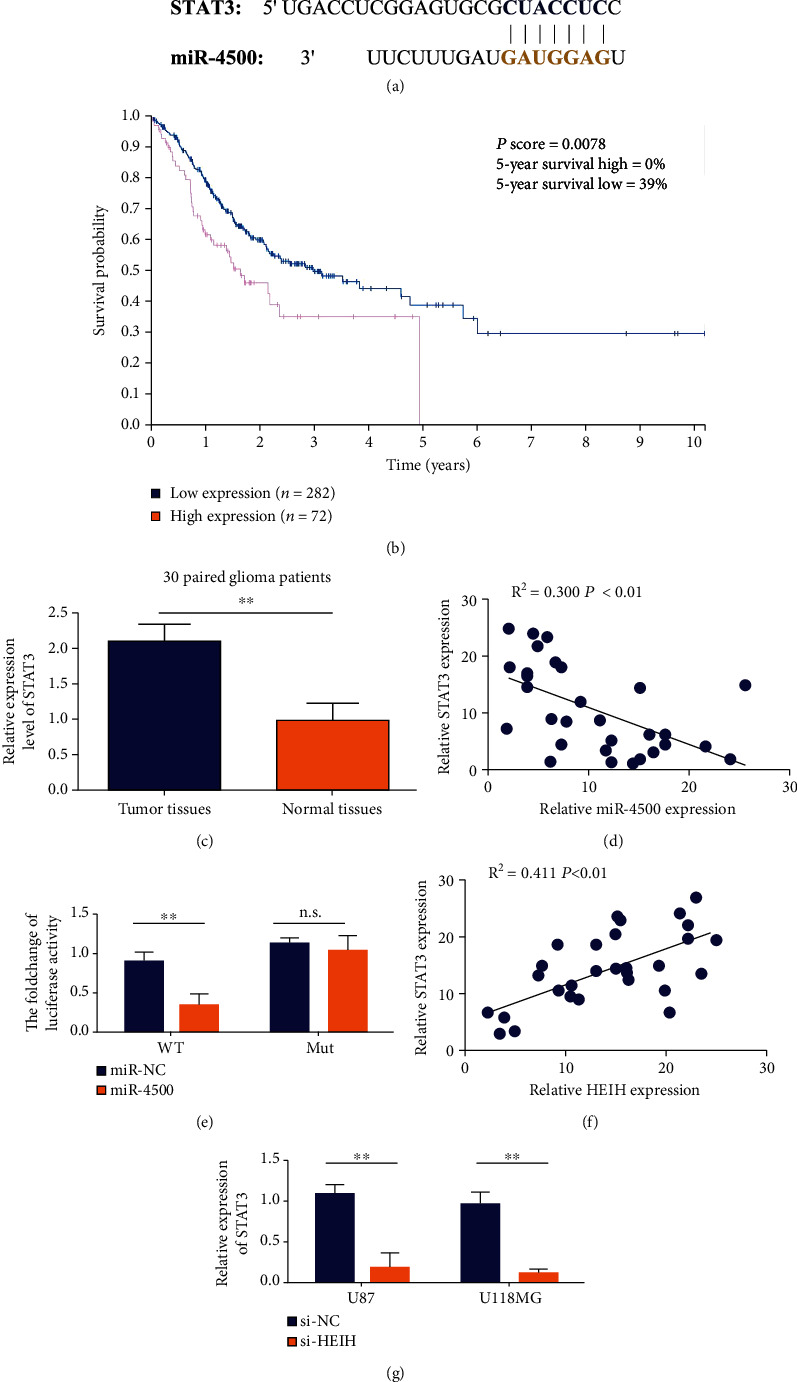
STAT3 is the downstream target of miR-4500. (a) The binding site of STAT3 and miR-4500. (b) Analysis of overall survival of TCGA gastric cancer patients based on STAT3 expression. (c) qRT-PCR detection of STAT3 mRNA levels in gastric cancer tissues and matched normal tissues (*n* = 30). (d) The correlation between the expression of STAT3 and miR-4500 in gastric cancer tissues and matched normal tissues (*n* = 30). (e) The dual luciferase reporter gene measures the direct binding of miR-4500 and STAT3 in MGC-803 cells. (f) Correlation between STAT3 and HEIH expression in gastric cancer tissue (*n* = 30). (g) Use qRT-PCR to detect the expression of STAT3 after HEIH-specific siRNA transfected into gastric cancer cells. ^∗^*P* < 0.05 and ^∗∗^*P* < 0.01.

Bioinformatics software TargetScan predicted that the target gene of miR-4500 might be STAT3. The miR-4500 seed region has complementary pairing sequences with the 3′ untranslated region of STAT3 gene (Figure 5(a)). The results of survival analysis showed that patients with higher HEIH expression had worse prognosis. It was suggested that HEIH upregulation was positively correlated with worse prognosis in gastric cancer (Figure 5(b)). STAT3 expression was upregulated in gastric cancer tissues compared with paracancer tissues (Figure 5(c)). The correlation results of STAT3 and miR-4500 expression in gastric cancer tissues and paired normal tissues showed negative coexpression of STAT3 and miR-4500 (Figure 5(d)). As shown in Figure 5(e), the relative luciferase activity of the wild-type+miR-4500 group was significantly lower than that of the wild-type+miR-NC group, indicating that miR-4500 could effectively inhibit the wild-type plasmid luciferase activity. The relative luciferase activity of the mutant+miR-4500 group was significantly higher than that of the wild-type+miR-4500 group. These results indicated that miR-4500 could not inhibit the mutant plasmid luciferase activity. Therefore, only miR-4500 can effectively bind to the 3′ UTR region of the wild-type plasmid. The correlation results of STAT3 and HEIH expression in gastric cancer tissue showed that STAT3 and HEIH showed coexpression positive correlation (Figure 5(f)). Real-time PCR results showed that STAT3 expression level in the si-HEIH group was significantly lower than that in the si-NC group, and the difference between the two groups was statistically significant (Figure 5(g)).

### 3.6. STAT3 Reversed the Effect of HEIH on Gastric Cancer

**Figure 6:**
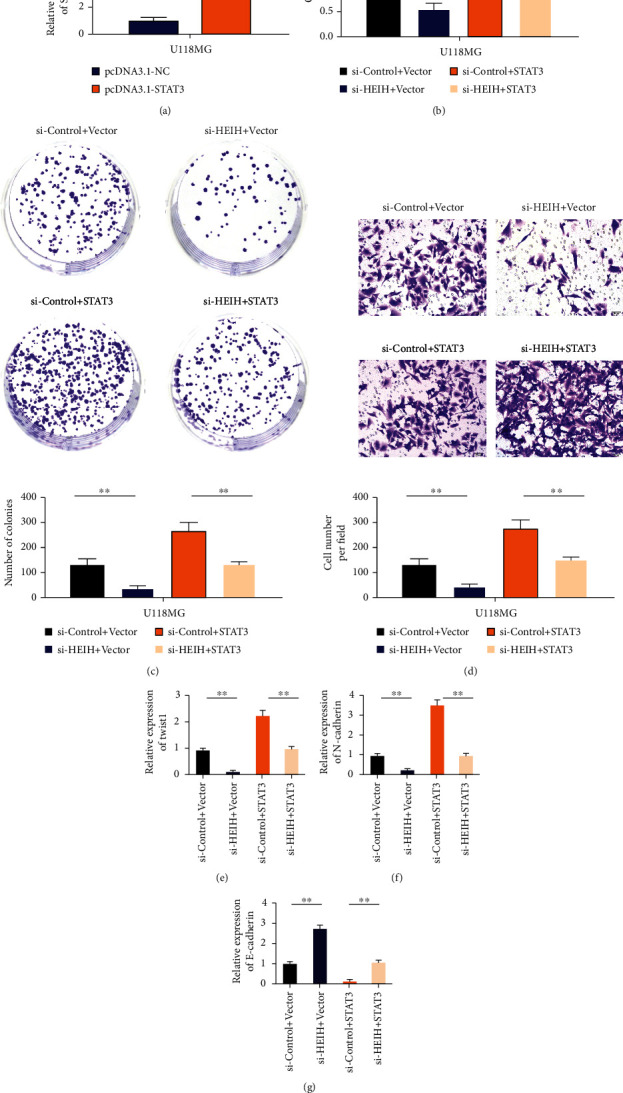
STAT3 reversed the effect of HEIH on gastric cancer. (a) Detection of transfection efficiency of STAT3 overexpression plasmid. (b) Detection of cell proliferation ability of BGC-823 cell line cotransfected with HEIH-specific siRNA or STAT3 plasmid. (c) Clone formation ability test of BGC-823 cell line cotransfected with HEIH-specific siRNA or STAT3 plasmid. (d) Detection of cell invasion ability of BGC-823 cell line cotransfected with HEIH-specific siRNA or STAT3 plasmid. (e) qRT-PCR detects the expression level of Twist1 in the cell line after cotransfection. (f) qRT-PCR detects the expression level of N-cadherin in the cell line after cotransfection. (g) qRT-PCR detects the expression level of E-cadherin in the cell line after cotransfection. ^∗^*P* < 0.05 and ^∗∗^*P* < 0.01.

Real-time PCR results showed that the expression level of STAT3 in the pcDNA3.1 STAT3 group was significantly higher than that in the NC group, with statistically significant difference between the two groups (Figure 6(a)). CCK8 results showed that compared with the si-HEIH+Vector-NC group, the cell activity of the si-HEIH+STAT3 cotransfected group was enhanced (Figure 6(b)). Compared with the si-HEIH+Vector-NC group, si-HEIH+STAT3 cotransfected cells had higher clonal formation and invasion ability (Figures 6(c) and 6(d)). After HEIH silencing, the expression of Twist1 and N-cadherin decreased. Meanwhile, after si-HEIH+STAT3 transfection, the expression levels of Twist1 and N-cadherin were upregulated (Figures 6(e) and 6(f)). The expression of E-cadherin increased after HEIH silencing. Meanwhile, after si-HEIH+STAT3 transfection, the expression of E-cadherin decreased (Figure 6(g)).

### 3.7. HEIH Inhibits Autophagy and Promotes Glycolysis

**Figure 7:**
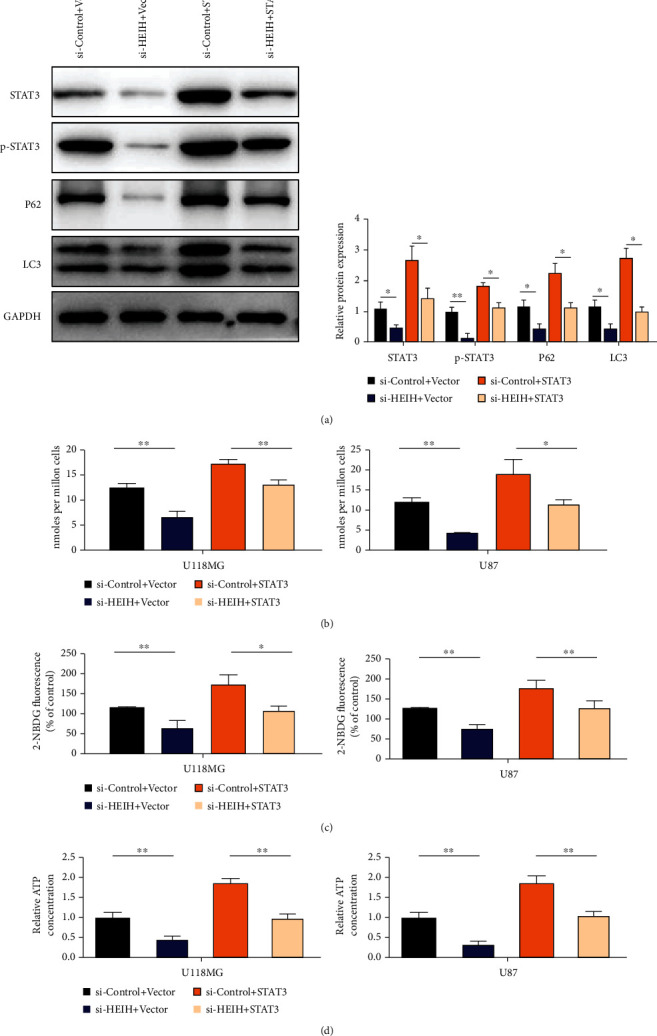
HEIH inhibits autophagy and promotes glycolysis. (a) Western blotting detects the protein levels of STAT3, p-STAT3, p62, and LC3 in treated BGC-823 cells. (b) Lactic acid production was detected in gastric cancer cells. (c) Gastric cancer cell glucose uptake detection. (d) ATP production was detected in gastric cancer cells. ^∗^*P* < 0.05 and ^∗∗^*P* < 0.01.

To further investigate the role of HEIH, we detected the protein levels of STAT3, p-STAT3, P62, and LC3 in the treated BGC-823 cells by Western blotting. The results showed that the protein levels of STAT3, p-STAT3, P62, and LC3 decreased after HEIH silencing. After si-HEIH+STAT3 transfection, the protein levels of STAT3, p-STAT3, P62, and LC3 were upregulated (Figure 7(a)). These results suggest that HIEH silencing can inhibit autophagy of gastric cancer cells. Subsequently, lactate production, glucose uptake, and ATP production in gastric cancer cells were measured after different treatments. The results showed that lactate production, glucose uptake, and ATP production decreased after HEIH addiction. These results showed that after HEIH silencing, glycolysis of gastric cancer cells was inhibited (Figures 7(b)–7(d)).

## 4. Discussion

The occurrence and development of gastric cancer is a malignant tumor with multigene mutation, multisignal pathway, and multifactor and multistep participation. Under the background of in-depth research on molecular targeted therapy of tumors and increasing clinical application, a large number of studies have shown that lncRNA is related to the occurrence and development of gastric cancer. Gastric juice lncRNAs have high specificity and can be used as biomarkers for the diagnosis and prognosis of gastric cancer [18]. lncRNAs in a single gastric juice are insufficiently sensitive as biomarkers although they are highly specific. More combined studies can be carried out, such as the combination of multiple gastric juice lncRNAs, the combination of gastric juice lncRNAs with plasma lncRNAs, and the combination of gastric juice lncRNAs with serum tumor markers to improve sensitivity [19]. But the specific sources and molecular mechanisms of lncRNAs in gastric juice need to be further explored.

lncRNA plays a dual role in malignant gastric cancer [17]. Li et al. [18] conducted cell transfection on the pathological tissues of 15 patients with malignant gastric cancer. Then, real-time quantitative reverse transcription polymerase chain reaction (RT-QPCR) analysis showed that NEAT1 expression was upregulated in malignant brain glial tissue. NEAT1 knockout can reduce the proliferation, invasion, and metastasis of tumor cells [19–21]. RNA-binding luciferase assay by immunoprecipitation confirmed that miRNA-449b-5p was bound to NEAT1 and acts as a “molecular sponge” to it. That is, the expression of miRNA-449b-5p can be negatively regulated. Shi et al. conducted statistical analysis on the expression of intergene-long noncoding gene H19 in 158 cases of gastric cancer combined with gastric cancer grade. The expression of H19 in high-grade gastric cancers was significantly higher than that in low-grade gastric cancers. Meanwhile, the expression level of H19 increased with the grade of gastric cancer. Inhibition of H19 expression by siRNA significantly reduced the invasion and migration ability of both malignant gastric cancer cells [22]. H19 may play a role in promoting the invasion and migration of gastric cancer cells as a precursor of miR-675. Ma et al. [23] analyzed the relationship between MALAT1 expression level and clinicopathological features of gastric cancer. The results showed that MALAT1 expression was increased in tumor tissues compared with adjacent normal brain tissues. The expression level was positively correlated with WHO grade and volume of gastric cancer. Overexpression of MALAT1 accelerates the growth of gastric cancer cells and enhances the ability of tumor cells to invade and metastasize. The mechanism may be that MALAT1 regulates the expression of genes related to cell metastasis and cell cycle [24, 25].

In order to investigate the clinical value of HEIH, qRT-PCR was used to confirm the high expression of HEIH in gastric cancer. In order to study the effect of HEIH on the biological function of gastric cancer cells, we downregulated and upregulated HEIH expression in gastric cancer cells. Subsequently, CCK8, clonogenesis, and Transwell assay were performed to determine the effects of HEIH on proliferation, clonogenesis, and invasion of gastric cancer cells. The results suggest that HEIH can regulate the malignant proliferation of gastric cancer cells. Mechanism studies have shown that upregulated HEIH binds miR-4500 to affect autophagy and glycolysis through the STAT3 signaling pathway and promote the proliferation and migration of gastric cancer cells. Autophagy and glucose metabolism reprogramming play an important role in tumor proliferation, drug resistance, invasion, and metastasis. Autophagy has been found to regulate glucose metabolism and affect the malignant progression of tumors.

Bioinformatics software TargetScan predicted that the target gene of miR-4500 might be STAT3. The miR-4500 seed region has complementary pairing sequences with the 3′ untranslated region of STAT3 gene. Overexpression of STAT3 can be detected in a variety of tumor tissues and cells. Previous studies have found that autophagy level changes in a variety of tumors at different stages of tumor genesis and development. Autophagy can promote and inhibit it in different ways [26, 27]. The interaction between STAT3 and autophagy is a complex process [12]. Our results suggest that HEIH can inhibit autophagy by regulating STAT3 in gastric cancer cell lines. One of the characteristics of glucose metabolism in tumor cells is the use of glycolysis even when oxygen content is normal, namely, the Warburg effect. The internal mechanism of Warburg effect is very complex, which may be related to oncogene activation, tumor suppressor gene inactivation, abnormal expression of glucose metabolism enzymes, and changes in tumor microenvironment. The specific mechanism needs to be further studied. In this study, HEIH can also promote glycolysis of gastric cancer cells by regulating STAT3.

## 5. Conclusion

This study confirmed that HEIH is upregulated in gastric cancer tissues. HEIH can promote malignant proliferation of gastric cancer cells and promote tumor proliferation and invasion in vitro. It is suggested that abnormally high expression of HEIH may be one of the factors of poor prognosis in patients with gastric cancer. HEIH may serve as a risk factor for shorter survival and higher risk of metastasis in patients in the future. This study will enrich the molecular mechanism of lncRNAs regulating the occurrence and development of gastric cancer and provide a new experimental basis for clinical diagnosis and treatment of gastric cancer. This study may also improve new candidate sites for pathological diagnosis and molecular drug therapy for future clinical treatment of gastric cancer.

## Data Availability

The data used to support the findings of this study are available from the corresponding author upon request.
